# Supplementary material for: The influence of maternal blood glucose during pregnancy on weight outcomes at birth and preschool age in offspring exposed to hyperglycemia first detected during pregnancy, in a South African cohort
Source: PLoS One. 2021 Oct 21;16(10):e0258894. doi: 10.1371/journal.pone.0258894 (PMC8530360; doi:10.1371/journal.pone.0258894)
Supplement: S1 File — (PDF) [file pone.0258894.s001.pdf]

The influence of maternal blood glucose during pregnancy on weight outcomes at birth and preschool age in offspring exposed to hyperglycemia first detected during pregnancy, in a South African cohort

Supplementary materials

Maternal BMI – blood glucose interaction analyses

S1 Fig – Birthweight z-score: Maternal BMI interaction with postprandial glucose

Source	SS	df	MS
Model	54.7725204	9	6.08583559
Residual	361.417401	317	1.14011798
Total	416.189922	326	1.2766562

Number of obs	=	327
F(9, 317)	=	5.34
Prob > F	=	0.0000
R-squared	=	0.1316
Adj R-squared	=	0.1069
Root MSE	=	1.0678

weight_z1	Coef.	Std. Err.	t	P>|t|	[95% Conf.	Interval]
OGTT_2hr_gluc	.0195877	.0297905	0.66	0.511	-.0390245	.0781998
FBG	.1018182	.0508315	2.00	0.046	.0018084	.2018279
OGTT_1hr_gluc	.0123499	.0105931	1.17	0.245	-.0084918	.0331915
postprand_gluc	.1520459	.3397299	0.45	0.655	-.5163644	.8204562
maternal_age	-.0081545	.0103879	-0.78	0.433	-.0285924	.0122835
maternal_BMI	.0127193	.0559148	0.23	0.820	-.0972917	.1227303
female_gender	.3583885	.1196747	2.99	0.003	.1229314	.5938455
caesarian_delivery	.0955991	.0622176	1.54	0.125	-.0268124	.2180106
postprand_gluc	0	(omitted)				
maternal_BMI	0	(omitted)				
c.postprand_gluc#c.maternal_BMI	.0013203	.0098472	0.13	0.893	-.0180538	.0206945
_cons	-2.215339	1.903882	-1.16	0.245	-5.961181	1.530502

S2 Fig – Birthweight z-score: Maternal BMI interaction with fasting blood glucose at HFDP diagnosis

Source	SS	df	MS
Model	56.002192	9	6.22246578
Residual	360.18773	317	1.13623889
Total	416.189922	326	1.2766562

Number of obs	=	327
F(9, 317)	=	5.48
Prob > F	=	0.0000
R-squared	=	0.1346
Adj R-squared	=	0.1100
Root MSE	=	1.0659

weight_z1	Coef.	Std. Err.	t	P>|t|	[95% Conf.	Interval]
OGTT_2hr_gluc	.0196394	.0297379	0.66	0.509	-.0388693	.078148
FBG	.2957913	.1922782	1.54	0.125	-.0825114	.674094
OGTT_1hr_gluc	.012195	.0105761	1.15	0.250	-.0086133	.0330032
postprand_gluc	.1854075	.0720662	2.57	0.011	.043619	.3271959
maternal_age	-.0086568	.0103301	-0.84	0.403	-.028981	.0116674
maternal_BMI	.0522317	.0315376	1.66	0.099	-.0098178	.1142812
female_gender	.3640002	.1195923	3.04	0.003	.1287053	.5992952
caesarian_delivery	.0956436	.0621082	1.54	0.125	-.0265528	.21784
FBG	0	(omitted)				
maternal_BMI	0	(omitted)				
c.FBG#c.maternal_BMI	-.0054408	.005187	-1.05	0.295	-.0156461	.0047645
_cons	-3.528787	1.183044	-2.98	0.003	-5.856397	-1.201176

S3 Fig – Birthweight z-score: Maternal BMI interaction with 2-hour blood glucose at HFDP diagnosis

Source	SS	df	MS
Model	54.8424092	9	6.09360102
Residual	361.347512	317	1.13989752
Total	416.189922	326	1.2766562

Number of obs	=	327
F(9, 317)	=	5.35
Prob > F	=	0.0000
R-squared	=	0.1318
Adj R-squared	=	0.1071
Root MSE	=	1.0677

weight_z1	Coef.	Std. Err.	t	P>|t|	[95% Conf.	Interval]
OGTT_2hr_gluc	-.0062741	.0967034	-0.06	0.948	-.1965356	.1839874
FBG	.0995883	.0509319	1.96	0.051	-.000619	.1997956
OGTT_1hr_gluc	.0123113	.010593	1.16	0.246	-.0085302	.0331527
postprand_gluc	.1977354	.071507	2.77	0.006	.0570472	.3384236
maternal_age	-.0084321	.0103537	-0.81	0.416	-.0288027	.0119386
maternal_BMI	.0129566	.0266616	0.49	0.627	-.0394994	.0654126
female_gender	.3570082	.1197411	2.98	0.003	.1214206	.5925958
caesarian_delivery	.0954931	.0622119	1.53	0.126	-.0269073	.2178936
OGTT_2hr_gluc	0	(omitted)				
maternal_BMI	0	(omitted)				
c.OGTT_2hr_gluc#c.maternal_BMI	.0007735	.002747	0.28	0.778	-.0046311	.0061782
_cons	-2.206348	1.074553	-2.05	0.041	-4.320504	-.0921921

S4 Fig– Preschool weight z-score: Maternal BMI interaction with postprandial glucose

Source	SS	df	MS
Model	23.9169306	9	2.65743673
Residual	285.059855	138	2.06565113
Total	308.976786	147	2.1018829

Number of obs	=	148
F(9, 138)	=	1.29
Prob > F	=	0.2495
R-squared	=	0.0774
Adj R-squared	=	0.0172
Root MSE	=	1.4372

weight_z2	Coef.	Std. Err.	t	P>|t|	[95% Conf.	Interval]
OGTT_2hr_gluc	.0316498	.0705225	0.45	0.654	-.1077947	.1710942
FBG	-.0255253	.112192	-0.23	0.820	-.2473629	.1963123
OGTT_1hr_gluc	.0014102	.0814658	0.02	0.986	-.1596724	.1624928
postprand_gluc	1.005283	.7088494	1.42	0.158	-.3963277	2.406893
maternal_age	.0171694	.022396	0.77	0.445	-.0271144	.0614532
maternal_BMI	.1295349	.1131214	1.15	0.254	-.0941404	.3532102
female_gender	.0942732	.2471437	0.38	0.703	-.394405	.5829513
caesarian_delivery	.1001557	.1235671	0.81	0.419	-.1441739	.3444853
postprand_gluc	0	(omitted)				
maternal_BMI	0	(omitted)				
c.postprand_gluc#c.maternal_BMI	-.0198947	.0199216	-1.00	0.320	-.0592858	.0194964
_cons	-6.997395	4.02958	-1.74	0.085	-14.9651	.9703077

S5 Fig – Preschool weight z-score: Maternal BMI interaction with fasting blood glucose at HFDP diagnosis

Source	SS	df	MS
Model	24.5261099	9	2.72512332
Residual	284.450676	138	2.06123678
Total	308.976786	147	2.1018829

Number of obs	=	148
F(9, 138)	=	1.32
Prob > F	=	0.2308
R-squared	=	0.0794
Adj R-squared	=	0.0193
Root MSE	=	1.4357

weight_z2	Coef.	Std. Err.	t	P>|t|	[95% Conf.	Interval]
OGTT_2hr_gluc	.0321125	.0704443	0.46	0.649	-.1071771	.1714022
FBG	.4108149	.3920844	1.05	0.297	-.3644549	1.186085
OGTT_1hr_gluc	-.0048823	.0814571	-0.06	0.952	-.1659478	.1561831
postprand_gluc	.2828735	.1463185	1.93	0.055	-.0064426	.5721896
maternal_age	.020143	.0225062	0.89	0.372	-.0243586	.0646446
maternal_BMI	.0795905	.0566429	1.41	0.162	-.0324096	.1915907
female_gender	.1346162	.2498542	0.54	0.591	-.3594213	.6286537
caesarian_delivery	.091928	.1235811	0.74	0.458	-.1524293	.3362854
FBG	0	(omitted)				
maternal_BMI	0	(omitted)				
c.FBG#c.maternal_BMI	-.0108117	.0095008	-1.14	0.257	-.0295977	.0079743
_cons	-5.516617	2.394026	-2.30	0.023	-10.25033	-.7829015

S6 Fig – Preschool weight z-score: Maternal BMI interaction with 2-hour blood glucose at HFDP diagnosis

Source	SS	df	MS			
				Number of obs	=	148
				F(9, 138)	=	1.20
Model	22.428054	9	2.492006	Prob > F	=	0.2998
Residual	286.548732	138	2.07644009	R-squared	=	0.0726
				Adj R-squared	=	0.0121
Total	308.976786	147	2.1018829	Root MSE	=	1.441

weight_z2	Coef.	Std. Err.	t	P>|t|	[95% Conf.	Interval]
OGTT_2hr_gluc	-.0733617	.2135244	-0.34	0.732	-.4955642	.3488408
FBG	-.0308468	.1152732	-0.27	0.789	-.258777	.1970834
OGTT_1hr_gluc	.000884	.081693	0.01	0.991	-.1606479	.162416
postprand_gluc	.3190279	.1451497	2.20	0.030	.0320228	.606033
maternal_age	.0158792	.0226268	0.70	0.484	-.0288609	.0606192
maternal_BMI	-.0097346	.0545711	-0.18	0.859	-.1176382	.098169
female_gender	.0716367	.250409	0.29	0.775	-.4234979	.5667712
caesarian_delivery	.0902043	.1249901	0.72	0.472	-.1569392	.3373477
OGTT_2hr_gluc	0	(omitted)				
maternal_BMI	0	(omitted)				
c.OGTT_2hr_gluc#c.maternal_BMI	.0030552	.0058252	0.52	0.601	-.0084629	.0145734
_cons	-2.097593	2.341547	-0.90	0.372	-6.727543	2.532356

S7 Fig – Preschool BMI z-score: Maternal BMI interaction with postprandial blood glucose

Source	SS	df	MS			
				Number of obs	=	138
				F(9, 128)	=	0.61
Model	11.8095475	9	1.31217194	Prob > F	=	0.7829
Residual	273.313693	128	2.13526323	R-squared	=	0.0414
				Adj R-squared	=	-0.0260
Total	285.123241	137	2.08119154	Root MSE	=	1.4613

child_bmi_z_score_5yr	Coef.	Std. Err.	t	P>|t|	[95% Conf.	Interval]
OGTT_2hr_gluc	.0299664	.0754726	0.40	0.692	-.119369	.1793017
FBG	.0199103	.1185601	0.17	0.867	-.2146811	.2545018
OGTT_1hr_gluc	-.017862	.0876021	-0.20	0.839	-.1911977	.1554737
postprand_gluc	.3511105	.7641165	0.46	0.647	-1.160824	1.863046
maternal_age	.0229245	.0237098	0.97	0.335	-.0239894	.0698384
maternal_BMI	.0584054	.1226592	0.48	0.635	-.1842967	.3011075
female_gender	.1560115	.2619385	0.60	0.552	-.3622785	.6743016
caesarian_delivery	.047902	.1294215	0.37	0.712	-.2081806	.3039846
postprand_gluc	0	(omitted)				
maternal_BMI	0	(omitted)				
c.postprand_gluc#c.maternal_BMI	-.0068763	.0215185	-0.32	0.750	-.0494544	.0357018
_cons	-3.4844	4.309324	-0.81	0.420	-12.01113	5.042334

S8 Fig– Preschool BMI z-score: Maternal BMI interaction with fasting blood glucose at HFDP diagnosis

Source	SS	df	MS
Model	11.6224964	9	1.29138849
Residual	273.500744	128	2.13672456
Total	285.123241	137	2.08119154

Number of obs	=	138
F(9, 128)	=	0.60
Prob > F	=	0.7915
R-squared	=	0.0408
Adj R-squared	=	-0.0267
Root MSE	=	1.4618

child_bmi_z_score_~r	Coef.	Std. Err.	t	P>|t|	[95% Conf.	Interval]
OGTT_2hr_gluc	.0313852	.0754056	0.42	0.678	-.1178176	.1805881
FBG	.07133	.4128103	0.17	0.863	-.7454856	.8881457
OGTT_1hr_gluc	-.0189696	.08808	-0.22	0.830	-.1932509	.1553116
postprand_gluc	.1083952	.1578591	0.69	0.494	-.2039561	.4207465
maternal_age	.023291	.0238136	0.98	0.330	-.0238282	.0704102
maternal_BMI	.0264731	.0596969	0.44	0.658	-.0916474	.1445936
female_gender	.1612947	.2650063	0.61	0.544	-.3630655	.6856549
caesarian_delivery	.0469023	.1294503	0.36	0.718	-.2092372	.3030419
FBG	0	(omitted)				
maternal_BMI	0	(omitted)				
c.FBG#c.maternal_BMI	-.0012004	.0099681	-0.12	0.904	-.0209239	.0185231
_cons	-2.431275	2.513542	-0.97	0.335	-7.404747	2.542196

S9 Fig – Preschool BMI z-score: Maternal BMI interaction with 2-hour blood glucose at HFDP diagnosis

Source	SS	df	MS
Model	12.0596262	9	1.33995846
Residual	273.063614	128	2.13330949
Total	285.123241	137	2.08119154

Number of obs	=	138
F(9, 128)	=	0.63
Prob > F	=	0.7713
R-squared	=	0.0423
Adj R-squared	=	-0.0250
Root MSE	=	1.4606

child_bmi_z_score_5yr	Coef.	Std. Err.	t	P>|t|	[95% Conf.	Interval]
OGTT_2hr_gluc	-.0665233	.2218621	-0.30	0.765	-.5055153	.3724687
FBG	.0117207	.1206514	0.10	0.923	-.2270088	.2504501
OGTT_1hr_gluc	-.0154447	.0877188	-0.18	0.861	-.1890113	.158122
postprand_gluc	.1216566	.1562245	0.78	0.438	-.1874602	.4307734
maternal_age	.0216308	.0238844	0.91	0.367	-.0256287	.0688902
maternal_BMI	-.0055819	.0560967	-0.10	0.921	-.1165787	.1054149
female_gender	.1374996	.2649435	0.52	0.605	-.3867364	.6617357
caesarian_delivery	.0379623	.1307971	0.29	0.772	-.2208421	.2967667
OGTT_2hr_gluc	0	(omitted)				
maternal_BMI	0	(omitted)				
c.OGTT_2hr_gluc#c.maternal_BMI	.0028077	.0059937	0.47	0.640	-.009052	.0146673
_cons	-1.232491	2.407793	-0.51	0.610	-5.996722	3.53174

S1 Table – Association between maternal blood glucose levels during pregnancy and offspring weight categories at birth

Birthweight status	RRR	Lower 95%CI	Upper 95%CI
SGA			
OGTT_2hr_gluc	0.91	0.67	1.23
FBG	0.61	0.30	1.23
OGTT_1hr_gluc	0.90	0.67	1.21
postprand_gluc	0.41	0.17	0.95
maternal_age	1.06	0.97	1.17
maternal_BMI	0.96	0.88	1.04
female_gender	0.34	0.11	1.11
caesarian_delivery	1.20	0.69	2.09
_cons	2025.27	1.14	3605294.00
AGA	(base outcome)		
LGA			
OGTT_2hr_gluc	1.06	0.93	1.21
FBG	1.08	0.87	1.34
OGTT_1hr_gluc	1.00	0.95	1.06
postprand_gluc	1.58	1.15	2.16
maternal_age	1.00	0.96	1.05
maternal_BMI	1.03	0.99	1.06
female_gender	1.46	0.86	2.48
caesarian_delivery	1.46	1.10	1.92
_cons	0.00	0.00	0.02

Abbreviations*: postprandial_gluc – Maternal 2-hour postprandial glucose during the third trimester*
OGTT_1hr_gluc – Maternal OGTT 1-hour glucose at HFDP diagnosis
OGTT_2hr_gluc - Maternal OGTT 2-hour glucose at HFDP diagnosis
Maternal_BMI – Maternal Body Mass Index at pregnancy booking
Maternal_age – Maternal age at pregnancy booking
FBG – Maternal fasting blood glucose at HFDP diagnosis
Caesarian_delivery – Caesarian delivery at birth

S10 Fig – Poisson regression for association between maternal blood glucose during pregnancy and LGA at birth

```
Iteration 0:   log likelihood = -187.91119
Iteration 1:   log likelihood = -187.90806
Iteration 2:   log likelihood = -187.90806

Poisson regression                              Number of obs     =        311
                                                LR chi2(8)        =      19.84
                                                Prob > chi2       =     0.0110
Log likelihood = -187.90806                     Pseudo R2         =     0.0501
```

lgabin	IRR	Std. Err.	z	P>|z|	[95% Conf.	Interval]
OGTT_2hr_gluc	1.033138	.0592266	0.57	0.570	.9233399	1.155993
FBG	1.035388	.0888642	0.41	0.685	.8750784	1.225065
OGTT_1hr_gluc	1.000303	.0252534	0.01	0.990	.9520122	1.051044
postprand_gluc	1.331619	.1642147	2.32	0.020	1.045707	1.695703
maternal_age	1.003912	.0194322	0.20	0.840	.9665393	1.042731
maternal_BMI	1.017637	.0136061	1.31	0.191	.9913157	1.044657
female_gender	1.279372	.2841842	1.11	0.267	.8277917	1.977299
caesarian_delivery	1.29566	.1538469	2.18	0.029	1.026642	1.63517
_cons	.0075544	.008234	-4.48	0.000	.0008921	.0639697

Note: _cons estimates baseline incidence rate.

S2 Table – Association between maternal blood glucose levels during pregnancy and offspring BMI categories at preschool age

BMI status	RRR Std. Err.	Lower 95%CI	Upper 95%CI
Normal	(base outcome)		
Underweight			
OGTT_2hr_gluc	1.04	0.78	1.41
FBG	1.06	0.66	1.71
OGTT_1hr_gluc	0.86	0.61	1.21
postprand_gluc	0.65	0.34	1.22
maternal_age	0.96	0.88	1.06
maternal_BMI	1.05	0.99	1.12
female_gender	0.49	0.18	1.38
caesarian_delivery	1.00	0.60	1.64
_cons	11.35	0.05	2567.97
Overweight___obese			
OGTT_2hr_gluc	1.02	0.79	1.32
FBG	1.12	0.76	1.64
OGTT_1hr_gluc	0.93	0.69	1.25
postprand_gluc	1.16	0.69	1.93
maternal_age	1.00	0.92	1.08
maternal_BMI	1.07	1.01	1.13
female_gender	0.89	0.37	2.13
caesarian_delivery	1.46	0.94	2.26
_cons	0.01	0.00	1.29

Abbreviations*: postprandial_gluc – Maternal 2-hour postprandial glucose during the third trimester*
OGTT_1hr_gluc – Maternal OGTT 1-hour glucose at HFDP diagnosis
OGTT_2hr_gluc - Maternal OGTT 2-hour glucose at HFDP diagnosis
Maternal_BMI – Maternal Body Mass Index at pregnancy booking
Maternal_age – Maternal age at pregnancy booking
FBG – Maternal fasting blood glucose at HFDP diagnosis
Caesarian_delivery – Caesarian delivery at birth

S11 Fig – Log-binomial regression for association between maternal blood glucose during pregnancy and overweight and obesity and overweight at preschool age

```
Iteration 0:   log likelihood = -120.41289  (not concave)
Iteration 1:   log likelihood =  -73.25359
Iteration 2:   log likelihood = -71.543049
Iteration 3:   log likelihood = -71.500526
Iteration 4:   log likelihood = -71.500041
Iteration 5:   log likelihood = -71.500041

Generalized linear models                         No. of obs      =        148
Optimization     : ML                             Residual df     =        139
                                                  Scale parameter =          1
Deviance         =  143.0000815                   (1/df) Deviance =   1.028778
Pearson          =  146.0130548                   (1/df) Pearson  =   1.050454

Variance function: V(u) = u*(1-u/1)               [Binomial]
Link function    : g(u) = ln(u)                   [Log]

                                                  AIC             =   1.087838
Log likelihood   = -71.50004073                   BIC             =  -551.6124
```

overw_bin_5yrs	Risk Ratio	OIM Std. Err.	z	P>|z|	[95% Conf.	Interval]
OGTT_2hr_gluc	.9495773	.0962664	-0.51	0.610	.7784614	1.158307
FBG	1.171748	.1592838	1.17	0.244	.897686	1.52948
OGTT_1hr_gluc	.9439075	.0988883	-0.55	0.582	.768694	1.159059
postprand_gluc	1.25097	.2050757	1.37	0.172	.9072073	1.724992
maternal_age	1.023221	.0304253	0.77	0.440	.965293	1.084625
maternal_BMI	1.015224	.0199014	0.77	0.441	.9769581	1.054989
female_gender	.8054235	.2630897	-0.66	0.508	.4246013	1.527803
caesarian_delivery	1.322753	.2332533	1.59	0.113	.9362213	1.86887
_cons	.0187502	.0273253	-2.73	0.006	.0010778	.3262059
