# Supplementary material for: The influence of maternal blood glucose during pregnancy on weight outcomes at birth and preschool age in offspring exposed to hyperglycemia first detected during pregnancy, in a South African cohort
Source: PLoS One. 2021 Oct 21;16(10):e0258894. doi: 10.1371/journal.pone.0258894 (PMC8530360; doi:10.1371/journal.pone.0258894)
Supplement: S1 Checklist — (PDF) [file pone.0258894.s003.pdf]

PRO2D Offspring - STROBE Statement—checklist of items that should be included in reports of observational studies

	Item No	Recommendation	Page No
**Title and abstract**	1	(*a*) Indicate the study's design with a commonly used term in the title or the abstract	Title page
		(*b*) Provide in the abstract an informative and balanced summary of what was done and what was found	Abstract
**Introduction**			
Background/rationale	2	Explain the scientific background and rationale for the investigation being reported	Introduction, paragraphs 1-5
Objectives	3	State specific objectives, including any prespecified hypotheses	Introduction, paragraph 6
**Methods**			
Study design	4	Present key elements of study design early in the paper	Methods, paragraph 1
Setting	5	Describe the setting, locations, and relevant dates, including periods of recruitment, exposure, follow-up, and data collection	Methods, paragraph 1
Participants	6	*Cross-sectional study*—Give the eligibility criteria, and the sources and methods of selection of participants	Methods, paragraph 1
Variables	7	Clearly define all outcomes, exposures, predictors, potential confounders, and effect modifiers. Give diagnostic criteria, if applicable	Methods - Outcomes<br><br>Methods – Data measured
Data sources/ measurement	8*	For each variable of interest, give sources of data and details of methods of assessment (measurement). Describe comparability of assessment methods if there is more than one group	Methods - Outcomes<br><br>Methods – Data measured
Bias	9	Describe any efforts to address potential sources of bias	Statistical data analysis
Study size	10	Explain how the study size was arrived at	Methods, Sample size
Quantitative variables	11	Explain how quantitative variables were handled in the analyses. If applicable, describe which groupings were chosen and why	Methods- statistical data analysis
Statistical methods	12	(*a*) Describe all statistical methods, including those used to control for confounding	Methods – statistical data analysis
		(*b*) Describe any methods used to examine subgroups and interactions	Not applicable
		(*c*) Explain how missing data were addressed	Statistical data analysis

(*d*) *Cohort study*—If applicable, explain how loss to follow-up was addressed<br>*Case-control study*—If applicable, explain how matching of cases and controls was addressed<br>*Cross-sectional study*—If applicable, describe analytical methods taking account of sampling strategy	Methods – sample size
(*e*) Describe any sensitivity analyses	

Continued on next page

**Results**			
Participants	13*	(a) Report numbers of individuals at each stage of study—eg numbers potentially eligible, examined for eligibility, confirmed eligible, included in the study, completing follow-up, and analysed	Results paragraph 1 and Fig 1
		(b) Give reasons for non-participation at each stage	Fig 1 – Study flow chart
		(c) Consider use of a flow diagram	Results - Fig 1 – Study flow chart
Descriptive data	14*	(a) Give characteristics of study participants (eg demographic, clinical, social) and information on exposures and potential confounders	Results – Table 1
		(b) Indicate number of participants with missing data for each variable of interest	Results – Table 1
		(c) *Cohort study*—Summarise follow-up time (eg, average and total amount)	Results Table 1
Outcome data	15*	*Cohort study*—Report numbers of outcome events or summary measures over time	Results – paragraph 2
		Case-control study—Report numbers in each exposure category, or summary measures of exposure	
		Cross-sectional study—Report numbers of outcome events or summary measures	
Main results	16	(*a*) Give unadjusted estimates and, if applicable, confounder-adjusted estimates and their precision (eg, 95% confidence interval). Make clear which confounders were adjusted for and why they were included	Results paragraph 4
		(*b*) Report category boundaries when continuous variables were categorized	Methods-Outcomes,
		(*c*) If relevant, consider translating estimates of relative risk into absolute risk for a meaningful time period	Not relevant
Other analyses	17	Report other analyses done—eg analyses of subgroups and interactions, and sensitivity analyses	Not applicable
**Discussion**			
Key results	18	Summarise key results with reference to study objectives	Discussion paragraph 1
Limitations	19	Discuss limitations of the study, taking into account sources of potential bias or imprecision. Discuss both direction and magnitude of any potential bias	Discussion last paragraph
Interpretation	20	Give a cautious overall interpretation of results considering objectives, limitations, multiplicity of analyses, results from similar studies, and other relevant evidence	Conclusion
Generalisability	21	Discuss the generalisability (external validity) of the study results	Discussion – limitations
**Other information**			

Funding	22	Give the source of funding and the role of the funders for the present study and, if applicable, for the original study on which the present article is based	Funding

*Give information separately for cases and controls in case-control studies and, if applicable, for exposed and unexposed groups in cohort and cross-sectional studies.

Note: An Explanation and Elaboration article discusses each checklist item and gives methodological background and published examples of transparent reporting. The STROBE checklist is best used in conjunction with this article (freely available on the Web sites of PLoS Medicine at http://www.plosmedicine.org/, Annals of Internal Medicine at http://www.annals.org/, and Epidemiology at http://www.epidem.com/). Information on the STROBE Initiative is available at www.strobe-statement.org.
